# Systemic lupus erythematosus extends beyond a type I interferonopathy, as demonstrated by NET‐activated monocytes

**DOI:** 10.1002/ctm2.70599

**Published:** 2026-02-08

**Authors:** Xiaolin Cao, Niels van Heusden, Daan K. J. Pieren, Bettina C. Geertsema‐Hoeve, Ellen D. Kaan, Patrick F. Greve, Maarten Limper, Marianne Boes

**Affiliations:** ^1^ Center for Translational Immunology University Medical Center Utrecht Utrecht University Utrecht the Netherlands; ^2^ Department of Rheumatology and Clinical Immunology University Medical Center Utrecht Utrecht University Utrecht the Netherlands; ^3^ Department of Internal Medicine Flevo Hospital Almere the Netherlands; ^4^ Department of Pediatrics University Medical Center Utrecht Utrecht University Utrecht the Netherlands

1

Dear Editor,

Type I interferon (IFN) signalling and neutrophil extracellular traps (NETs) formation are the primary contributors to the pathogenesis of systemic lupus erythematosus (SLE).^1^, ^2^ Anifrolumab, a recently approved monoclonal antibody prescribed for moderate to severe SLE,^3^ selectively inhibits type I IFN signalling but demonstrates incomplete efficacy in many patients.^4^ We found that NET‐activated monocytes release CCL5 and promote CD4+ T cell proliferation independently of type I IFN signalling, underscoring a distinct, NET‐driven mechanism in SLE that functions outside canonical type I IFN pathway. Our data highlight the need for complementary therapies targeting NETs in patients unresponsive to anifrolumab treatment.

The chemokine CCL5 (RANTES), a potential biomarker elevated in SLE patients,^5^ may contribute to disease progression by mediating leukocytes chemotaxis^6^ or potentially exerting a previously unrecognized immunostimulatory function. We confirmed that plasma CCL5 concentrations were notably elevated in seven SLE patients in remission than in healthy donors (HD) (Figure 1A). In a 2‐year longitudinal study, we screened nine active SLE patients, excluding patients who developed antiphospholipid syndrome.^7^ CCL5 levels were measured and plotted alongside Systemic Lupus Erythematosus Disease Activity Index (SLEDAI) scores (Figure 1B) or flare status (Figure 1C) for a representative case (Pt. 06), with same analyses shown for the other included patients (Figures S1 and S2). Across the cohort, CCL5 levels normalized to individual baselines were elevated during periods of high SLEDAI (Figure 1D,E) and flares (Figure 1F), supporting its potential as a dynamic disease activity marker. To further explore the functional axis of CCL5, we assessed the expression of its highest‐affinity receptor CCR5, on monocytes from SLE patients and healthy controls. Monocyte subsets were identified as shown in Figure 1G. While the overall distribution of monocyte subclasses was comparable between HD and SLE groups (Figure S3A), monocytes from SLE patients exhibited a notable reduction in CD16 expression (Figure 1H).^8^ Monocytes from SLE patients displayed higher surface expression of CCR5 compared to those from healthy donors (Figure 1I,J), particularly within the pro‐inflammatory classical and intermediate subsets (Figure 1K). Within individual donors, higher plasma CCL5 levels are associated with increased CCR5 expression on monocytes (Figure 1L).

**FIGURE 1:**
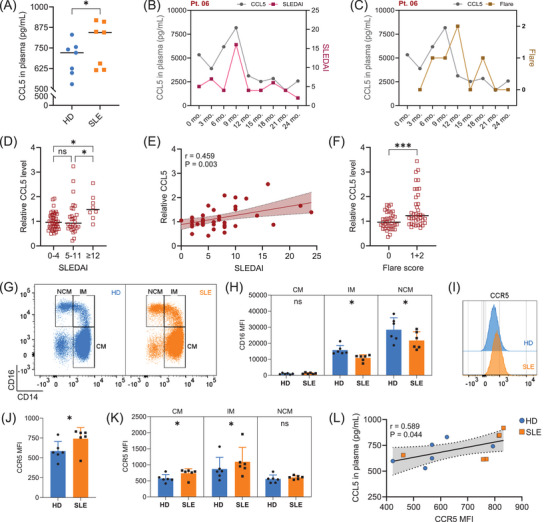
Plasma CCL5 levels are increased in systemic lupus erythematosus (SLE) patients compared with healthy donors and correlate with disease activity and flare severity. Consistently, monocytes from SLE patients exhibit elevated CCR5 expression relative to healthy donors. (A). CCL5 concentrations in plasma from healthy donors and SLE patients in remission (*n *= 7 per group, age‐ and gender‐matched). Wilcoxon test was applied. (B). Grouped plot of CCL5 plasma levels and SLE Disease Activity Index (SLEDAI) scores in a representative patient (Pt. 06). (C). Grouped plot of CCL5 plasma levels and flare status in Pt. 06. (D). Relative plasma CCL5 levels normalized to individual baseline (defined as the average of timepoints with SLEDAI ≤ 4 for each patient). Kruskal–Wallis test was used to compare relative CCL5 levels across three SLEDAI groups. (E). Correlation between relative CCL5 levels and SLEDAI scores across all patients. For each patient, relative CCL5 levels were averaged across the time points corresponding to each distinct SLEDAI score. Pearson correlation coefficient (r) and P‐value are shown. (F). Relative plasma CCL5 levels normalized to individual baseline (defined as the average CCL5 level when flare = 0). Flare scoring: 0 = no flare; 1 = mild flare; 2 = severe flare. A Mann–Whitney U test was applied to compare relative CCL5 levels between the no flare (0) and flare (1, 2) groups (A, D and F) Data presented as scatter dot plots with lines at median. (G). Representative flow cytometry plots showing CD14 and CD16 expression on monocytes from healthy donors and SLE patients. Classical monocytes (CM), intermediate monocytes (IM) and non‐classical monocytes (NCM). (H). CD16 expression on CM, IM and NCM in HD and SLE patients. Wilcoxon test was applied. (I). Representative histogram comparing CCR5 surface expression on monocytes from HD and SLE patients. (J). Quantification of CCR5 surface expression on monocytes in HD and SLE groups. Wilcoxon test was applied. (K). CCR5 expression on monocyte subsets (CM, IM and NCM) from HD and SLE patients. Wilcoxon test was applied. Mean fluorescence intensity (MFI) indicates marker surface expression. Data represent mean ± SEM from six donors. (L). Correlation between plasma CCL5 levels and monocyte CCR5 expression in six SLE patients in remission and six age‐ and gender‐matched healthy donors. Pearson correlation coefficient (r) and P‐value are shown. Each dot represents one donor. Statistical significance was defined as follows: 0.1234 (ns), 0.0332 (*), 0.0021 (**), 0.0002 (***), < 0.0001 (****). The samples used in panels A and G‐L were obtained from the INTERFERON cohort (see Table S1), while the samples used in panels B to F were derived from the PROFILE cohort (see Table S2).

To investigate the mechanisms driving CCL5/CCR5 upregulation in SLE, we mimicked the disease environment in vitro by stimulating monocytes from healthy donors with IFNα and NETs induced by LPS‐activated platelet.^1^, ^9^ We found that IFNα and NETs synergistically regulate monocyte responses: CCL5 mRNA expression is upregulated by combined IFNα and NETs stimulation (Figure 2B), while CCL5 protein secretion is primarily induced by NETs in a DNA‐dependent manner (Figure 2C–E). Conversely, CCR5 mRNA is increased by IFNα alone (Figure 2F), but CCR5 surface protein expression on monocytes is maximally enhanced by the combined effect of IFNα and NETs (Figure 2G). Additionally, IFNα stimulation selectively increases CD80 expression on monocytes (Figure S4A,B), suggesting a potential for increased monocyte‐mediated T cell activation. To test whether CCL5 directly influenced T cell responses, we stimulated purified T cells with recombinant CCL5. Although CCL5 increased CCR5 expression on CD4+ T cells (Figure 2I), it did not promote their proliferation (Figure 2J) or activation markers expression (Figure 2K–M), indicating that additional signals are required to elicit functional T cell responses.

**FIGURE 2:**
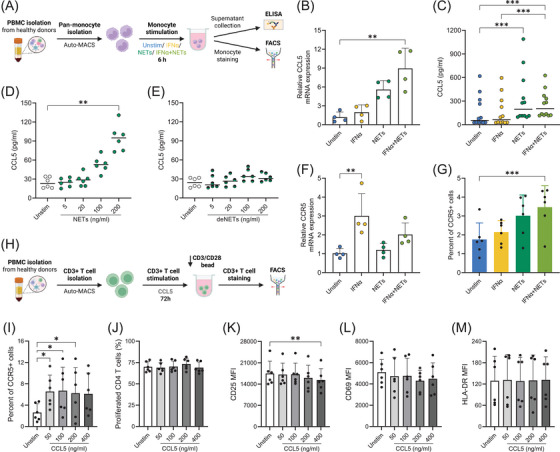
Interferon alpha (IFNα) and/or neutrophil extracellular trap (NET) exposure stimulates CCL5 and CCR5 upregulation in healthy monocytes; CCL5 does not directly stimulate CD4+ T cell activation or proliferation. (A). Experimental design: Negatively selected monocytes were cultured for 6 h under four conditions: medium alone (unstimulated), IFNα (100 U/mL), NETs (200 ng/mL), or a combination of IFNα and NETs. Following stimulation, culture supernatants were collected for CCL5 ELISA, and cells were harvested for extracellular staining and analysis by flow cytometry. (B). Relative CCL5 mRNA expression measured by Q‐PCR (mean ± SEM, *n* = 4). (C). CCL5 secretion in monocyte culture supernatants measured by ELISA (median indicated, *n* = 12). (D). Dose‐dependent CCL5 secretion in response to increasing concentrations of NETs (median indicated, *n* = 6). (E). CCL5 secretion after stimulation with DNase‐digested NETs (deNETs), containing approximately one‐third of the DNA content of intact NETs (median indicated, *n* = 6). (F). CCR5 mRNA expression in monocytes (mean ± SEM, *n* = 4). (G). Percentage of CCR5‐positive monocytes among total monocytes analysed by flow cytometry (mean ± SEM, *n* = 6). (H). Experimental design: Purified CD3+ T cells were cultured for 72 h with increasing concentrations of recombinant human CCL5 (0, 50, 100, 200 and 400 ng/mL) in the presence of CD3/CD28 activating beads at a ratio of 50 000 beads (1.25 µL) per 1 × 10^6^ T cells. (I). CCR5 surface expression on CD4+ T cells after CCL5 stimulation. (J). CD4+ T cell proliferation measured by CellTrace Violet (CTV) dilution after CCL5 treatment. Expression of activation markers CD25 (K), CD69 (L), and HLA‐DR (M) on CD4+ T cells following CCL5 stimulation. MFI represents mean fluorescence intensity. Data represent mean ± SEM from six donors. Statistical significance was defined as follows: 0.1234 (ns), 0.0332 (*), 0.0021 (**), 0.0002 (***), < 0.0001 (****). The Friedman test with Dunn's multiple comparisons test was applied to all comparisons.

Given that CCL5 alone does not activate T cells, we investigated whether IFNα‐ and/or NET‐stimulated monocytes could promote CD4+ T cell activation and proliferation. To approximate physiological conditions in PBMCs culture, monocyte‐derived dendritic cells (moDCs) were generated and stimulated with IFNα and/or NETs (Figure 3A). After 24 h, T cell activation markers were analysed, and proliferation was assessed following 9 days of culture. Our data showed that IFNα exhibited anti‐proliferative effects on CD4+ T cells,^9^ while NETs enhanced proliferation (Figure 3B) and HLA‐DR expression of CD4+ T cells (Figure S5E). CD25 expression was downregulated by IFNα (Figure 3C) and CD69 was upregulated by both stimuli (Figure 3D). Subsequently, isolated monocytes and autologous CD3+ T cells were co‐cultured without or with the presence of anifrolumab following monocyte pre‐stimulation with IFNα and/or NETs. NETs consistently induced CD4+ T cell proliferation, which was not inhibited by anifrolumab (Figure 3G). Anifrolumab enhanced CD25 expression in the presence of IFNα (Figure 3H) and fully abrogated IFNα‐induced upregulation of CD69 (Figure 3I) and IFNAR1 (Figure 3J). These data support that NET‐matured monocytes/moDCs enhance CD4+ T cell proliferation and that this NET‐driven activation is resistant to IFNAR blockade.

**FIGURE 3:**
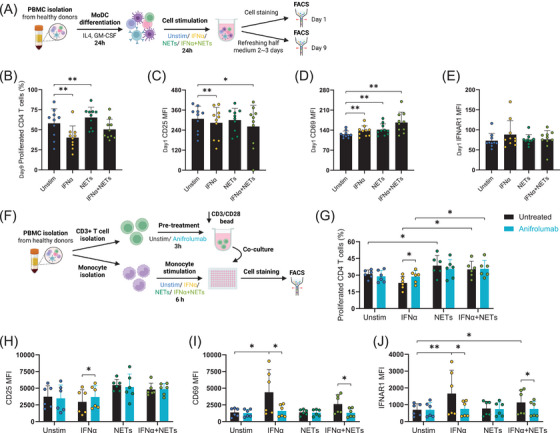
Anifrolumab does not inhibit NETs‐induced CD4+ T cell activation and proliferation (co‐cultures with moDC/monocytes). (A). Schematic representation of the experimental workflow corresponding to plots B–E. Monocytes within PBMCs were differentiated with IL‐4 and GM‐CSF for 24 h, then PBMC cultures were stimulated with IFNα and/or NETs for another 24 h. Cells were either analysed for activation markers at 24 h or maintained in IL2, IL7 and IL15 supplemented medium to assess CD4+ T cell proliferation at day 9. (B). CD4+ T cell proliferation after 9 days, assessed by CellTrace Violet (CTV) using flow cytometry. CD25 (C), CD69 (D) and IFNAR1 (E) expression on CD4+ T cells after 24 h of stimulation, measured by flow cytometry. MFI = mean fluorescence intensity. Data represent mean ± SEM from 10 donors. (F). Overview of the experimental workflow for monocyte and T cell co‐culture corresponding to the results shown in panels G–J. Monocytes and autologous CD3+ T cells were isolated from the same donor. Monocytes were pre‐stimulated with IFNα and/or NETs for 6 h, while CD3+ T cells were stained with CTV and activated using CD3/CD28 activating beads at a ratio of 50 000 beads (1.25 µL) per 1 × 10^6^ T cells, in the presence or absence of anifrolumab. The pre‐stimulated monocytes and activated T cells were then combined and co‐incubated for 72 h. (G). CD4+ T cell division profiles at 72 h under indicated stimulations. (H). CD25 expression on CD4+ T cells in monocyte‐T cell coculture after 72 h. (I). CD69 expression on CD4+ T cells in monocyte‐T cell coculture after 72 h. (J). IFNAR1 expression on CD4+ T cells in monocyte‐T cell coculture after 72 h. MFI = mean fluorescence intensity. Data represent mean ± SEM from six donors. Statistical significance was defined as follows: 0.1234 (ns), 0.0332 (*), 0.0021 (**), 0.0002 (***), < 0.0001 (****). Pairwise Wilcoxon tests were performed for each column pair, and P‐values were adjusted using the Bonferroni method to account for multiple comparisons.

To examine the direct influences of IFNα and NETs on T cells or monocytes, we stimulated each cell type separately with IFNα and/or NETs, with or without anifrolumab involved (Figure 4A). IFNα exposure attenuated proliferation of CD4+ T cells (Figure 4B)^10^ and reduced CD25 expression (Figure 4C), both of which were reversed by anifrolumab presence. IFNα increased CD69 and IFNAR1 surface expression, which was blocked by anifrolumab. These IFNα‐driven changes corroborate our co‐culture findings (Figure 3G–J). The NET‐driven enhancement of CD4+ T cell proliferation is mediated indirectly via monocyte activation, substantiating the role of NET‐induced monocyte phenotypic modulation in facilitating T cell proliferation. To further characterize the direct phenotypic and functional changes of monocytes in prolonged stimulation of IFNα and/or NETs, we cultured monocytes for up to 36 h. IFNα induced upregulation of maturation markers CD80 (Figure 4H), CD86 (Figure 4I), HLA‐ABC (Figure S6A) and HLA‐DR (Figure S6B), which were all significantly reduced by anifrolumab treatment. In contrast, NETs did not induce observable changes in these markers, suggesting that NET‐activated monocytes adopt a distinct activation state. Supporting this, cytokine analysis of monocyte supernatants revealed that NETs selectively increased CCL2 (Figure S6C), IL‐6 (Figure S6D), and IL‐1β (Figure S6G). The CCL2 increase was abolished by anifrolumab, while IL‐6 and IL‐1β were unaffected, other cytokines, including IFNγ (E), TNFα (F), and IL‐8 (H), were unchanged. These results suggest that NETs activate monocytes differently from IFNα, producing a cytokine profile that may enhance CD4+ T cell proliferation. Besides, NETs robustly triggered CCL5 secretion by monocytes, which was unaffected by IFNAR blockade (Figure 4J).

**FIGURE 4:**
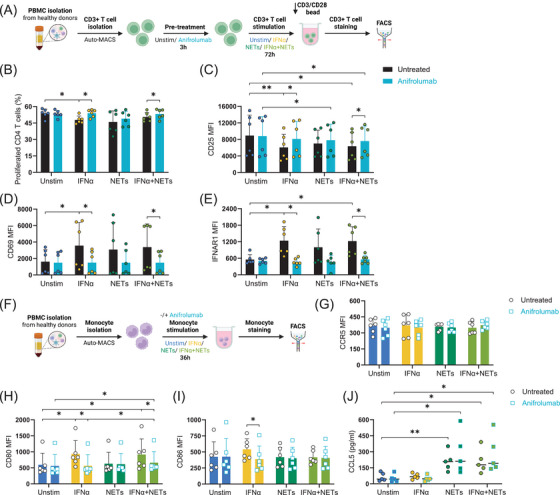
Anifrolumab pretreatment does inhibit IFNα‐mediated effects on monocyte and T cell activation, but not NETs‐mediated CCL5 secretion of monocytes. (A). Schematic of the experimental workflow to plots B‐E. CD3+ T cells isolated from healthy donors were stained with CellTrace Violet (CTV) and cultured for 72 h with IFNα and/or NETs in the presence of CD3/CD28 activating beads. To assess the role of type I IFN signalling, parallel cultures were treated with the IFNAR1‐blocking antibody anifrolumab. (B). Percentage of proliferated CD4+ T cells after 72 h of culture with IFNα, NETs, IFNα+ NETs, with or without anifrolumab. (C). CD25 expression on CD4+ T cells at 72 h. (D). CD69 expression on CD4+ T cells at 72 h. (E). IFNAR1 expression on CD4+ T cells at 72 h. MFI = mean fluorescence intensity. Data represent mean ± SEM from six donors. (F). Monocytes were isolated and stimulated with IFNα and/or NETs for 36 h, in the presence or absence of anifrolumab, then analysed by flow cytometry, as shown in plots G–I. (G). CCR5 expression on monocytes. (H). CD80 expression on monocytes. (I). CD86 expression on monocytes. MFI = mean fluorescence intensity. Data represent mean ± SEM from six donors. (J). CCL5 secretion by monocytes (as measured by ELISA), scatter dot lined at median (*n* = 5). Statistical significance was defined as follows: 0.1234 (ns), 0.0332 (*), 0.0021 (**), 0.0002 (***), < 0.0001 (****). Wilcoxon tests were performed for all column pairs and corrected for multiple comparisons.

In conclusion, our study identifies that NET‐activated monocytes promote CCL5 production and CD4+ T cell proliferation in SLE via a pathway independent of type I IFN signalling. While anifrolumab effectively suppresses IFNα‐mediated monocyte and T cell activation, it does not inhibit NET‐induced responses. Elevated plasma CCL5 levels correlate with disease activity, suggesting that CCL5 reflects ongoing inflammatory activity within a broader cytokine network driven NET‐activated monocyte. These findings suggest that persistent activation of monocytes by NETs may contribute to the limited efficacy of anifrolumab in certain SLE patients. By emphasizing the role of type I IFN‐independent, monocyte‐driven inflammatory pathways, our study underscores the need for complementary therapeutic strategies targeting both type I IFN signalling and NET‐activated myeloid responses. These findings also encourage further investigation into the molecular mechanisms underlying NET‐induced monocyte activation, including in vivo validation and extended functional studies, which may improve outcomes in patients with type I IFN‐independent or anifrolumab‐refractory disease.

## Supporting information



Supporting information

Supporting information

Supporting information

Supporting information

Supporting information

Supporting information

Supporting information

Supporting information

## References

[1] Lood C , Blanco LP , Purmalek MM , et al. Neutrophil extracellular traps enriched in oxidized mitochondrial DNA are interferogenic and contribute to lupus‐like disease. Nat Med. 2016;22(2):146‐153. doi:10.1038/nm.4027 26779811 10.1038/nm.4027 PMC4742415

[2] Garcia‐Romo GS , Caielli S , Vega B , et al. Netting neutrophils are major inducers of type I IFN production in pediatric systemic lupus erythematosus. Sci Transl Med. 2011;3(73):73ra20‐73ra20. doi:10.1126/scitranslmed.3001201 10.1126/scitranslmed.3001201 PMC3143837 21389264

[3] Furie R , Khamashta M , Merrill JT , et al. Anifrolumab, an anti–interferon‐α receptor monoclonal antibody, in moderate‐to‐severe systemic lupus erythematosus. Arthritis Rheumatol. 2017;69(2):376‐386. doi:10.1002/art.39962 28130918 10.1002/art.39962 PMC5299497

[4] Gatto M , Zen M , Cruciani C , Iaccarino L , Doria A . Navigating the landscape of SLE treatment: an expert viewpoint on the rationality and limitations of early biologic intervention. Autoimmun Rev. 2024;23(10):103612. doi:10.1016/j.autrev.2024.103612 39218330 10.1016/j.autrev.2024.103612

[5] Lit LC , Wong CK , Tam LS , Li EK , Lam CW . Raised plasma concentration and ex vivo production of inflammatory chemokines in patients with systemic lupus erythematosus. Ann Rheum Dis. 2006;65(2):209‐215. doi:10.1136/ard.2005.038315 15975968 10.1136/ard.2005.038315 PMC1798029

[6] Rawat K , Tewari A , Li X , et al. CCL5‐producing migratory dendritic cells guide CCR5+ monocytes into the draining lymph nodes. J Exp Med. 2023;220(6). doi:10.1084/jem.20222129 10.1084/jem.20222129 PMC10072223 36946983

[7] Patsouras MD , Sikara MP , Grika EP , Moutsopoulos HM , Tzioufas AG , Vlachoyiannopoulos PG . Elevated expression of platelet‐derived chemokines in patients with antiphospholipid syndrome. J Autoimmun. 2015;65:30‐37. doi:10.1016/j.jaut.2015.08.001 26283469 10.1016/j.jaut.2015.08.001

[8] Burbano C , Vasquez G , Rojas M . Modulatory effects of CD14+CD16++ monocytes on CD14++CD16− monocytes: a possible explanation of monocyte alterations in systemic lupus erythematosus. Arthritis Rheumatol. 2014;66(12):3371‐3381. doi:10.1002/art.38860 25168844 10.1002/art.38860

[9] Clark SR , Ma AC , Tavener SA , et al. Platelet TLR4 activates neutrophil extracellular traps to ensnare bacteria in septic blood. Nat Med. 2007;13(4):463‐469. doi:10.1038/nm1565 17384648 10.1038/nm1565

[10] Crouse J , Kalinke U , Oxenius A . Regulation of antiviral T cell responses by type I interferons. Nat Rev Immunol. 2015;15(4):231‐242. doi:10.1038/nri3806 25790790 10.1038/nri3806

