# Supplementary material for: Systemic lupus erythematosus extends beyond a type I interferonopathy, as demonstrated by NET‐activated monocytes
Source: Clin Transl Med. 2026 Feb 8;16(2):e70599. doi: 10.1002/ctm2.70599 (PMC12883034; doi:10.1002/ctm2.70599)
Supplement: Supplementary file 8 — Supporting information [file CTM2-16-e70599-s005.docx]

Letter to the Journal with previous submitted manuscript ID CTM2-2025-10-2814.

# Materials and Methods:

## **Recruitment of participants**

This project involved the analysis of peripheral blood and plasma samples obtained from both healthy donors and SLE patients. Peripheral blood from healthy participants was collected through the Mini Donor Service (MDS) at the University Medical Center (UMC) Utrecht. All participants provided written informed consent for the use of their samples in scientific research. The MDS works in accordance with the Code of Ethics of the World Medical Association (Declaration of Helsinki).

Peripheral blood samples anticoagulated with lithium heparin were acquired from SLE patients in sustained remission who attended the outpatient clinic of Department of Rheumatology and Clinical Immunology at UMC Utrecht. These patients consented to have additional blood drawn for experimental purposes alongside routine clinical sampling, in accordance with the INTERFERON-study protocol (protocol number NL47151.041.13). A total of seven plasma samples and six PBMC samples from this cohort were included in the present study.

Additionally, plasma samples collected in sodium citrate tubes from nine patients with active SLE were included from the PROFILE-study (protocol number NL75276.041.21). This longitudinal study collected blood samples every three months over a two-year period, yielding nine samples per patient across different time points. All samples were obtained with approval from the Medical Research Ethics Committee in Utrecht, the Netherlands. All patients signed a consent form prior to enrollment.

## **SLE disease activity**

SLE disease activities and flares were assessed using the SELENA- SLEDAI Flare Index. The definition of a severe flare is as follows: 1) increase of SLEDAI by >12; and/or 2) new/worse CNS involvement, vasculitis, glomerulonephritis, myositis, platelet counts <60,000/mm3, hemolytic anemia (hemoglobin <70 g/L), requiring doubling of the prednisone dose or a dose >0.5 mg/kg; and/or 3) need for hospitalization due to SLE; and/or 4) any manifestation requiring prednisone >0.5 mg/kg or new immunosuppressive therapy; and/or 4) increase in physician’s global assessment (PGA) to >2.5.

Based on the flare index, flare 0, flare 1 and flare 2 correspond to no flare, mild flare, and severe flare, respectively.

## **Cell Isolation and culture**

Peripheral blood was collected from donors into sodium citrate- anticoagulated tubes. Monocytes and T cells were isolated using an autoMACS Separator (Miltenyi Biotec, Cat. No. 130-092-545) according to the manufacturer’s instructions.

Cells were cultured in RPMI 1640 medium (Gibco, Cat. No. 52400-025) supplemented with 10% (v/v) fetal bovine serum (FBS) (Sigma-Aldrich, Cat. No. F7524), 1% (v/v) Penicillin- Streptomycin (Merck, Cat. No. P0781-100ML), and 1% (v/v) L-glutamine (Thermo Fisher Scientific, Cat. No. 25030024). Cultures were maintained at 37 °C in a humidified incubator with 5% CO₂.

## **Isolation and stimulation of monocytes**

Pan monocytes, including classical, intermediate, and non-classical subsets, were isolated by negative selection using the Pan Monocyte Isolation Kit (Miltenyi Biotec, Cat. No. 130-096-537) for stimulation experiments.

For co-culture experiments, CD14+ monocytes were isolated using anti-human CD14 microbeads (Miltenyi Biotec, Cat. No. 130-050-201) following the manufacturer’s instructions.

Isolated monocytes were stimulated by 100 U/mL IFNα (universal type I IFN, Bio-Techne, Cat. No. 11200-1) and/or 200 ng/mL *in vitro* generated NETs for 6 hours or 36 hours. To inhibit type I interferon signaling, monocytes were treated with 10 µg/mL anifrolumab (Saphnelo, AstraZeneca).

## **Isolation, activation and stimulation of T cells**

CD3+ T cells were isolated using human CD3 microbeads (Miltenyi Biotec, Cat. No. 130-050-101). Cells were activated and expanded with Dynabeads Human T-Activator CD3/CD28 (Gibco, Cat. No. 11161D) at a ratio of 50,000 beads (1.25 µL) per 1 × 10⁶ T cells.

For proliferation tracking, T cells were resuspended at 2 × 10⁶ cells/mL and stained with CellTrace Violet (CTV) (Fisher Scientific, Cat. No. 10220455) at a 1:2,000 dilution (v/v) for 30 minutes at room temperature and then washed with FBS prior to culture.

CD3+ T cells were stimulated with recombinant human CCL5 (0, 50, 100, 200, and 400 ng/mL; Cat. No. 278-RN-010/CF, Bio-Techne), 100 U/mL IFNα (universal type I IFN; Cat. No. 11200-1, Bio-Techne), and/or 200 ng/mL *in vitro*- generated NETs for 72 hours. To inhibit type I interferon signaling, CD3+ T cells were pre-treated with 10 µg/mL anifrolumab (Saphnelo, AstraZeneca), which remained present throughout the 72-hour culture period.

## **Differentiation of monocyte-derived dendritic cells (moDCs).**

PBMCs were isolated from freshly drawn blood and stained with CTV, then cultured with 100 ng/mL IL-4 (ImmunoTools, Cat. No. 11340045) and 100 ng/mL GM-CSF (ImmunoTools, Cat. No. 11343125) for 24 hours to induce monocyte differentiation. Cells were then washed once and further stimulated with 100 U/mL IFNα (universal type I IFN, Bio-Techne, Cat. No. 11200-1) and/or 200 ng/mL NETs for 24 hours. After stimulation, half of the cells were harvested and stained for activation markers, while the remaining cells were maintained in culture with fresh medium replenished every 2- 3 days. The culture medium was supplemented with 10 U/mL IL-2 (Novartis, RVG No. 13354), 10 ng/mL IL-7 (PeproTech, Cat. No. 200-07-10UG), and 10 ng/mL IL-15 (ImmunoTools, Cat. No. 11340153).

## **Production and quantification of NETs**

Neutrophils were isolated from peripheral blood using density gradient centrifugation followed by red blood cell lysis. Purified neutrophils were resuspended in DMEM/F‑12 medium (no phenol red, Gibco, Cat. No. 11039021), and seeded onto cell culture flasks (Greiner Bio-One, Cat. No. 658175), allowing adherence at 37 °C for 30 minutes.

Platelets were isolated separately from EDTA-anticoagulated whole blood and stimulated with 1×10⁵ EU/mL lipopolysaccharide (LPS) from Escherichia coli O111:B4 Merck, Cat. No. LPS25) for 1 hour at 37 °C. The activated platelets were then added to the adherent neutrophil layer and co-cultured for an additional hour to induce NETosis.

Following neutrophil extracellular trap (NET) induction, samples were treated with micrococcal nuclease (5 U/mL; Thermo Fisher Scientific, Cat. No. 88216) to digest chromatin and release NETs into the supernatant. The supernatant was collected and centrifuged at 1,000 ×g for 5 min to remove intact cells and debris. This step was repeated, and the clarified supernatant was transferred to a fresh tube for downstream analysis.

NETs were quantified by staining extracellular DNA with SYTOX™ Orange (0.2 µM; Invitrogen, Cat. No. 10338062), followed by fluorescence measurement. DNA concentrations were determined using a standard curve generated from serial dilutions of purified human genomic DNA (Promega, Cat. No. G3041).

Residual LPS in NET preparations was quantified using the Pierce™ Chromogenic Endotoxin Quant Kit (Thermo Fisher Scientific, Cat. No. A39552S), the manufacturer’s instructions was modified. To generate the standard curve, the kit-provided endotoxin standard (lyophilized E. coli O111:B4) was replaced with the same LPS used for NET induction, LPS from E. coli O111:B4 (Merck, Cat. No. LPS25). LPS standards were prepared in endotoxin-free water and then serially diluted 10-fold to generate a standard curve, with a range of 1 EU/mL to 0.001 EU/mL, including 0 EU/mL, based on an estimated activity of ~10 EU/ng as determined by the manufacturer’s chromogenic assay. NET samples and LPS standards were incubated with Limulus amebocyte lysate (LAL) reagent at 37 °C for 12 min. Chromogenic substrate was then added, and the reaction was stopped after 6 min with 25% acetic acid. Absorbance was measured at 405 nm using a microplate reader. Endotoxin concentrations in NET samples were calculated by comparison with the LPS standard curve and are reported as EU/mL.

## **Enzyme Linked Immunosorbent Assay (ELISA)**

Quantification of CCL5 was performed using the ELISA MAX™ Deluxe Set Human CCL5/RANTES (BioLegend, Cat. No. 440804), following the manufacturer’s instructions. All reagents and procedures were prepared and carried out according to the accompanying technical data sheet. Absorbance was measured at 450 nm using a microplate reader, and cytokine concentrations were calculated based on a standard curve generated from known concentrations of recombinant CCL5.

Cytokines secreted by NETs-stimulated monocytes were measured using the LEGENDplex™ Multi-Analyte Flow Assay Kit (BioLegend, Cat. No. 740808) following the manufacturer’s instructions. Briefly, culture supernatants were collected after stimulation for 48 hour and incubated with fluorescently labeled beads conjugated to capture antibodies for multiple cytokines. After washing, detection antibodies and streptavidin-PE were added, and the samples were analyzed by flow cytometry. Cytokine concentrations were calculated by comparison to standard curves provided in the kit.

Buffer dilutions were prepared using Dulbecco’s Phosphate-Buffered Saline (DPBS) (Sigma, Cat. No. D8537) and Aqua ad iniectabilia (sterile water, B. Braun Petzold, Cat. No. 143883).

## **Flow cytometry**

Cells were harvested and stained for viability using eFluor 780 viability dye (eBioscience, Cat. No. 65-0865-18) in phosphate-buffered saline (PBS) for 15 minutes at 4 °C. Following staining, cells were washed with 1% Bovine Serum Albumin (BSA) Fraction V (Roche, Cat. No. 10735094001) prepared in Dulbecco’s PBS (DPBS). Cells were then incubated with a cocktail of fluorochrome-conjugated antibodies for 30 minutes at 4 °C in the dark. After staining, cells were washed twice with 1% BSA buffer to remove unbound antibodies.

Flow cytometric analysis of cell surface marker expression was performed on a BD LSRFortessa™ Cell Analyzer (BD Biosciences, Cat. No. 647794), and data acquisition and analysis were conducted using BD FACSDiva software.

## **Quantitative PCR (Q-PCR)**

Monocytes were lysed, and total RNA was extracted using the RNeasy Mini Kit (Qiagen, Cat. No. 7404) according to the manufacturer’s instructions. RNA concentration and purity were measured using a NanoPhotometer® N60 (Implen, Cat. No. N60-Touch-IMP).

Complementary DNA (cDNA) synthesis was performed with the iScript cDNA Synthesis Kit (Bio-Rad, Cat. No. 1708891). The Q-PCR reactions were prepared in a total volume of 15 µL, containing 7.5 µL of SYBR Select Master Mix (Fisher Scientific, Cat. No. 13256519), 1 µL of combined forward and reverse primers (5 µM each), 3.5 µL of nuclease-free water (ddH₂O), and 2 µL of cDNA template (2.5 ng/µL).

Reactions were loaded onto a MicroAmp™ Fast Optical 96-Well Reaction Plate (Applied Biosystems, Cat. No. 4346907) and run on a QuantStudio™ 12K Flex Real-Time PCR System (Applied Biosystems, SN 285880344). Data acquisition and analysis were performed using the instrument’s software.

## **Statistical analysis and visualization**

Data analysis and visualization were performed using Microsoft Excel (version 2302), GraphPad Prism (version 10.1.2), FlowJo (version 10.8.1), and Adobe Illustrator (2022). Appropriate non-parametric statistical tests were applied based on data distribution and study design.

For two-group comparisons, the Wilcoxon matched-pairs signed rank test was used for paired data, and the Mann- Whitney U test for unpaired data. For comparisons involving multiple groups, the Friedman test was applied to paired datasets, while the Kruskal- Wallis test was used for unpaired datasets.

Correlations between variables were assessed using simple linear regression, and 95% confidence intervals for the regression lines were plotted to visualize the variability. The Pearson correlation coefficient (r) and corresponding p-values were reported for each analysis.

Statistical significance was defined as follows: 0.1234 (ns), 0.0332 (*), 0.0021 (**), 0.0002 (***), <0.0001 (****).

# Supplementary Figure 1 Figure 1. Individual plots of patient data used collectively in Figure 1D, E.


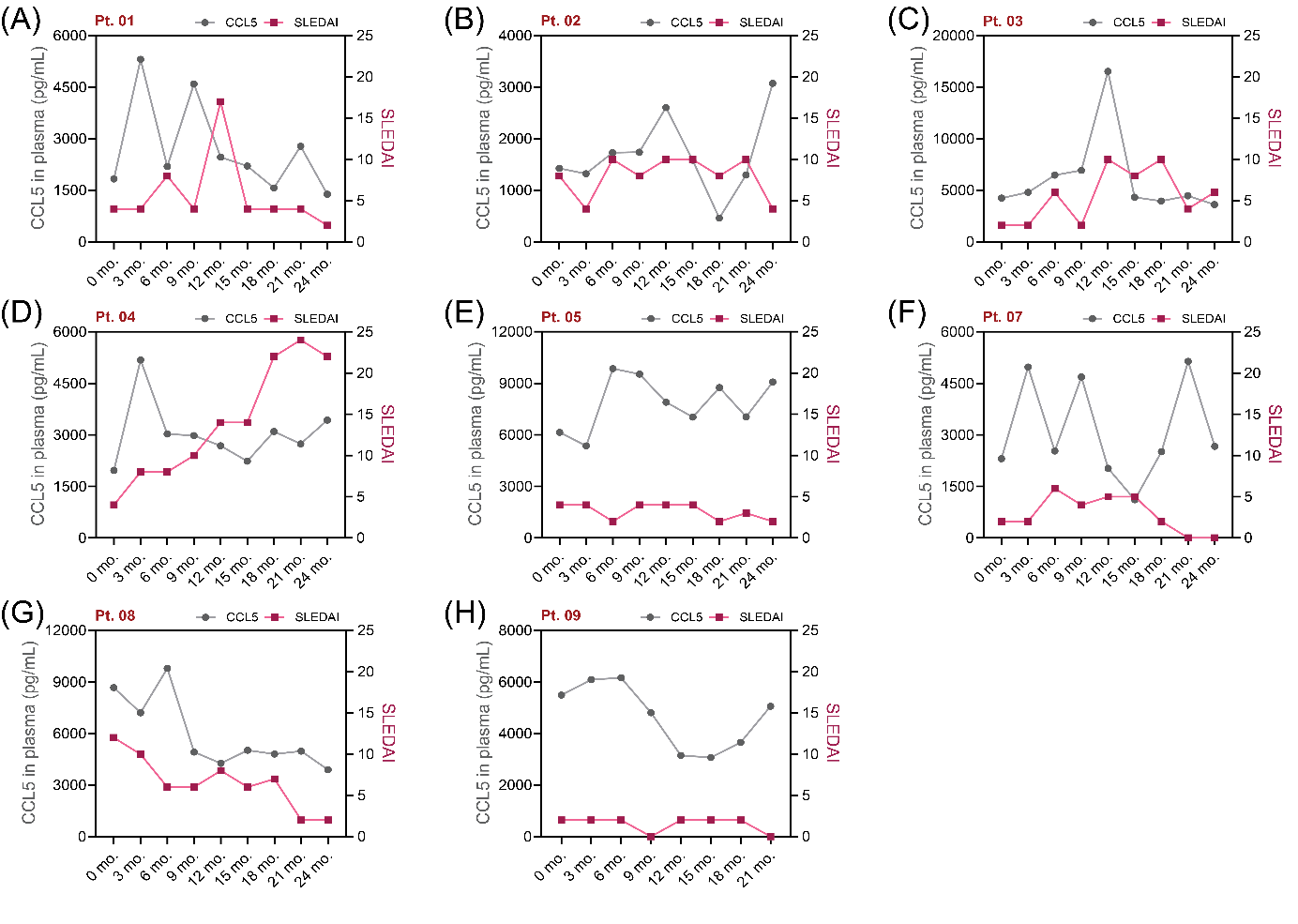


Grouped plot of SLEDAI and CCL5 plasma levels in (A). patient 01; (B). patient 02; (C). patient 03; (D). patient 04; (E). patient 05; (F). patient 07; (G). patient 08; (H). patient 09.

# Supplementary Figure 2 Individual plots of patient data used collectively in Figure 1F.


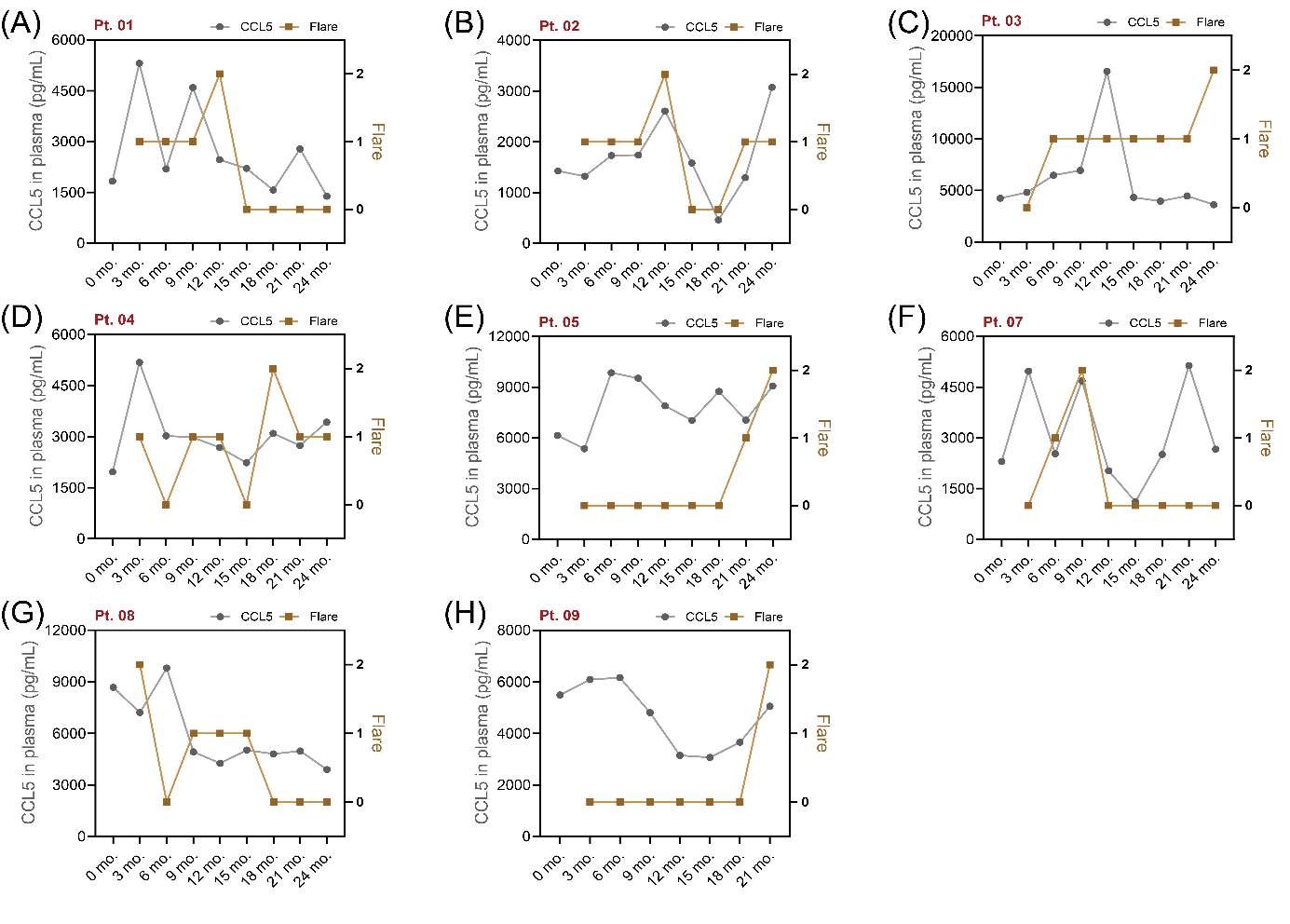


Grouped plot of flares and CCL5 plasma levels in (A). patient 01; (B). patient 02; (C). patient 03; (D). patient 04; (E). patient 05; (F). patient 07; (G). patient 08; (H). patient 09.

# Supplementary Figure 3 Marker expression on monocytes from SLE patients and healthy controls, and monocytes from healthy donors stimulated with IFNα and/or NETs.


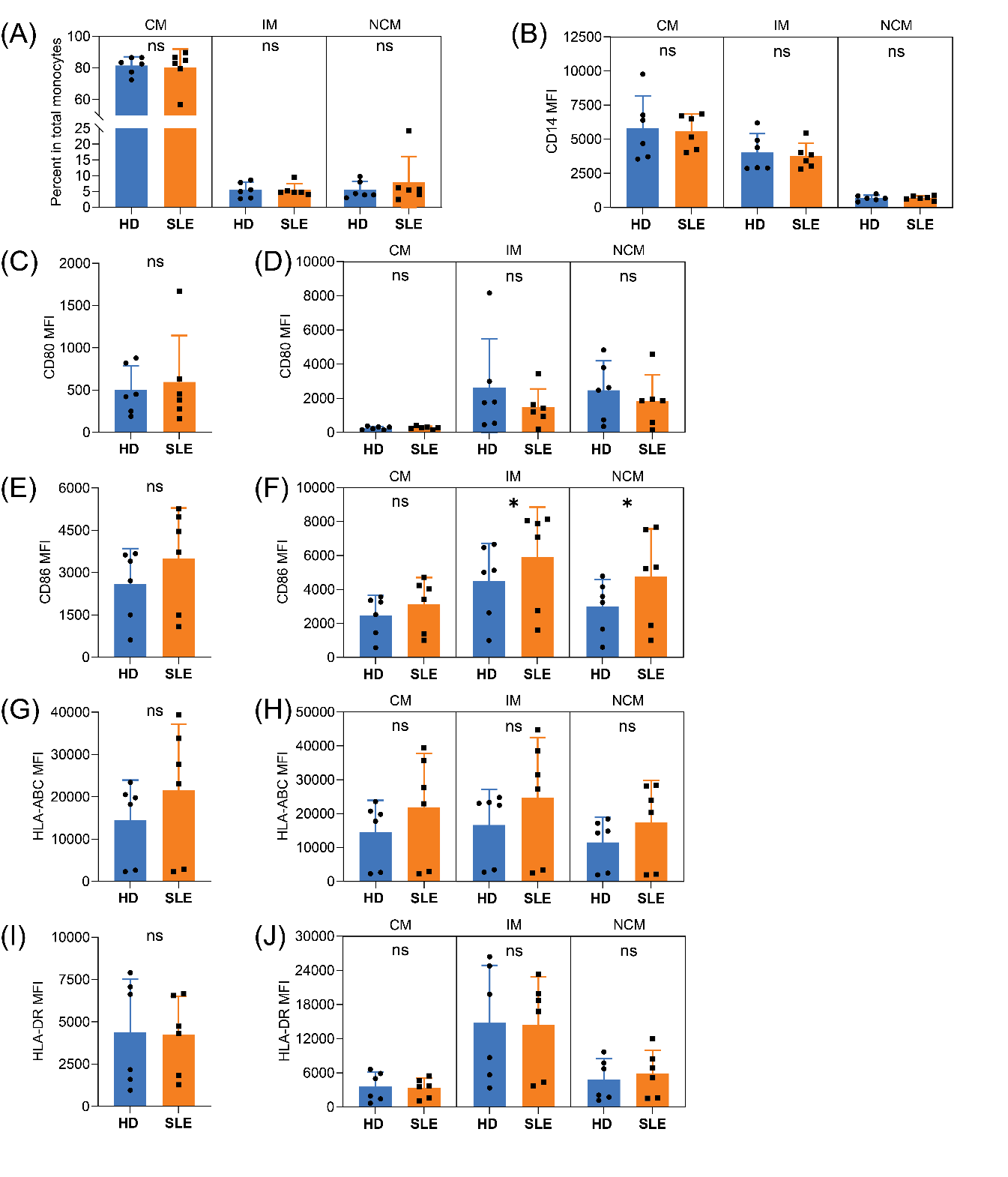


(A). Comparisons of subclass percentages among all monocytes between healthy donors and SLE patients. (B). CD14 expression on classical, intermediate and non-classical monocytes among healthy donors and SLE patients. (C). CD80 surface expression on monocytes of HD and SLE patients. (D). CD80 surface expression on monocyte subclasses of HD and SLE patients. (E). CD86 surface expression on monocytes of HD and SLE patients. (F). CD86 surface expression on monocyte subclasses of HD and SLE patients. (G). HLA-ABC surface expression on monocytes of HD and SLE patients. (H). HLA-ABC surface expression on monocyte subclasses of HD and SLE patients. (I). HLA-DR surface expression on monocytes of HD and SLE patients. (J). HLA-DR surface expression on monocyte subclasses of HD and SLE patients. MFI represents mean fluorescence intensity. Data represent mean ± SEM from six donors. Wilcoxon tests were applied.

# Supplementary Figure 4 Quantification of NETs preparations and residual LPS amount. IFNα induces CD80 and CD86 expression on monocytes.


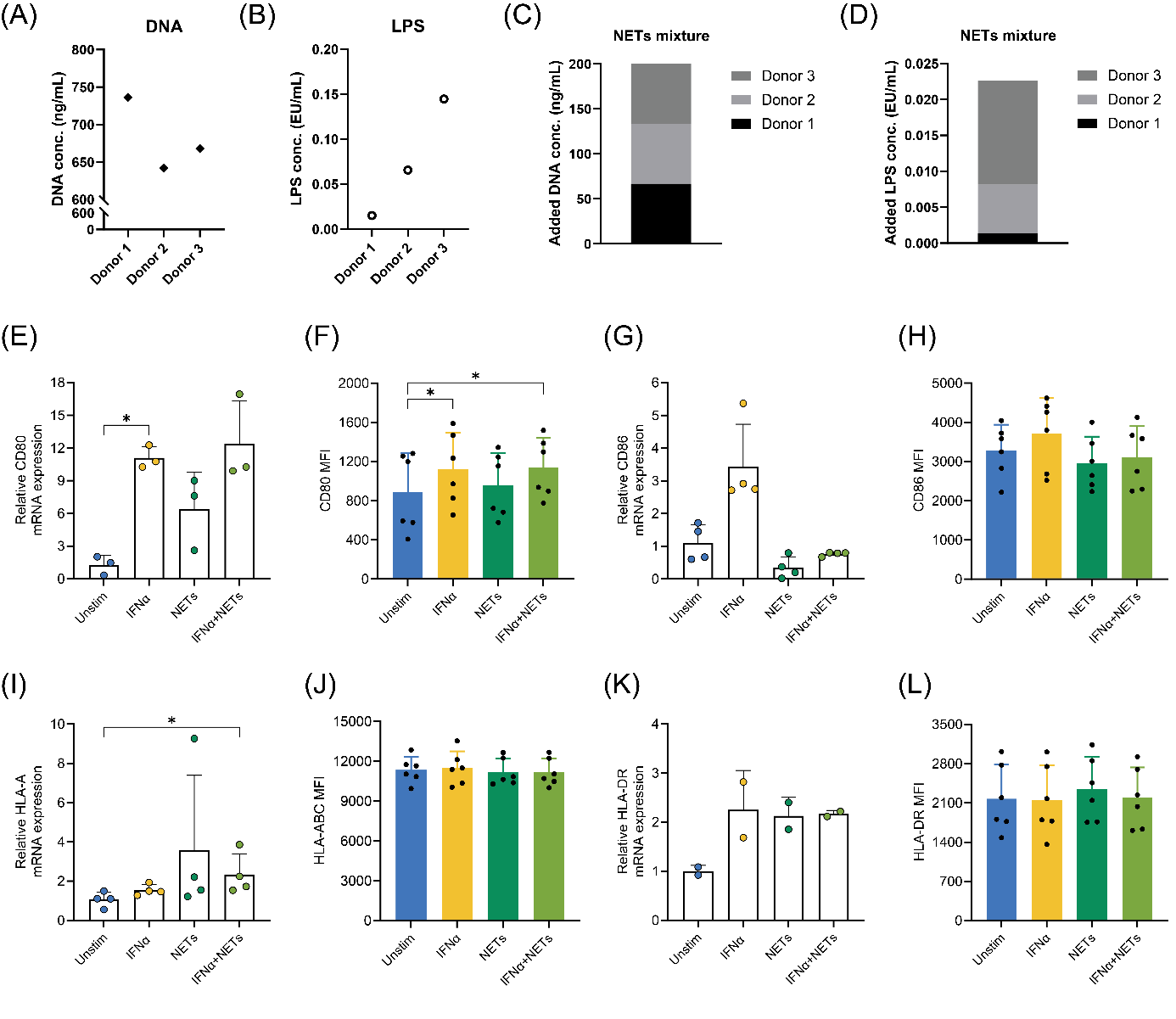


NETs were isolated from three donors and analyzed for DNA and LPS content. (A) DNA concentration (ng/mL) in NETs from each individual donor. (B) LPS concentration (EU/mL) in NETs from each donor, indicating the level of endotoxin contamination. (C) For stimulation experiments, NETs were pooled in equal amounts (66.67 ng/mL from each donor) to achieve a final DNA concentration of approximately 200 ng/mL. (D) The contribution of LPS from each donor to the final NETs mixture is shown as a stacked column; the combined amount of residual LPS in the NETs was 0.0226 EU/mL. E-L: Expression of co-stimulatory and HLA molecules in monocytes after 6 hours stimulation. (E). CD80 mRNA expression. (F). CD80 protein expression (MFI). (G). CD86 mRNA expression. (H). CD86 protein expression (MFI). (I). HLA-A mRNA expression. (J). HLA-ABC protein expression (MFI). (K). HLA-DR mRNA expression. (L). HLA-DR protein expression (MFI). Data represent mean ± SEM from six donors. MFI, mean fluorescence intensity. Friedman tests were applied.

# Supplementary Figure 5 Markers expression on CD4+ T cells (co-cultures with moDC/monocytes).


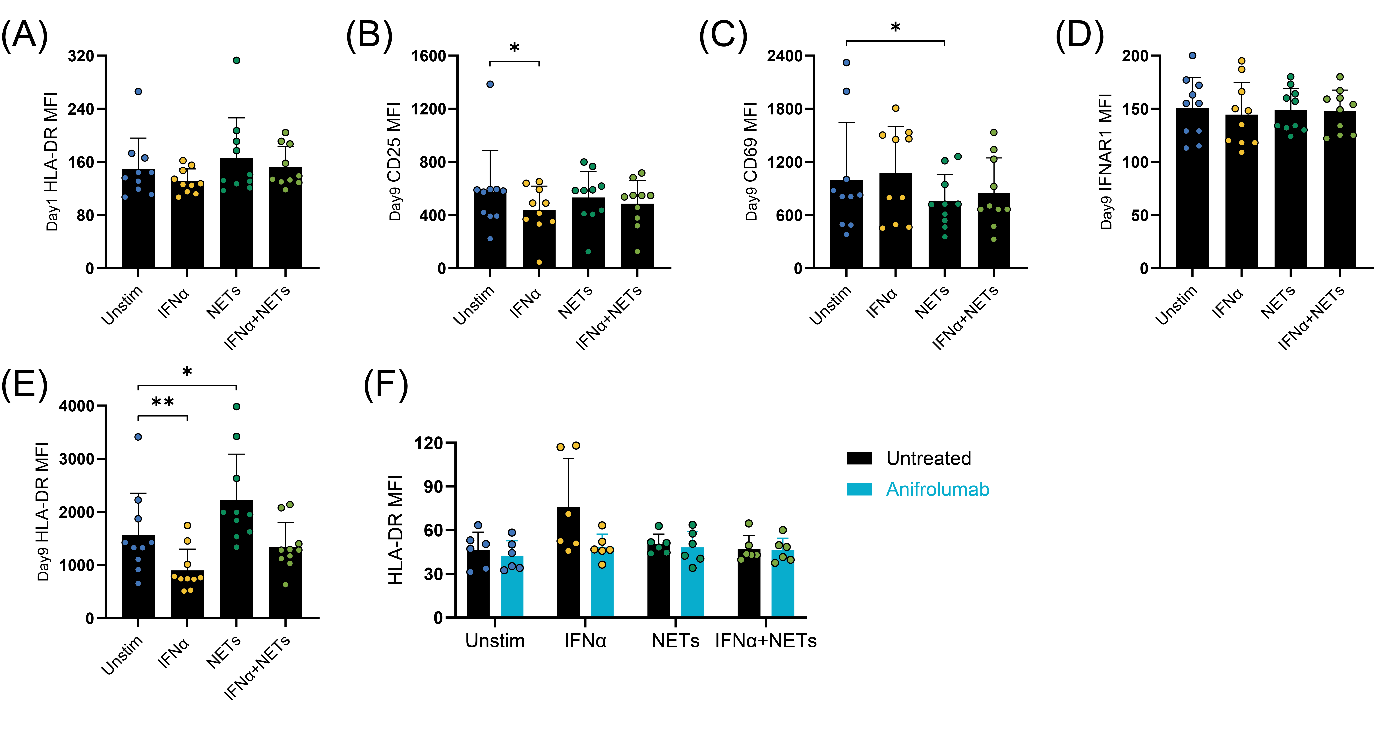


(A). Day 1 HLA-DR expression on CD4+ T cells in moDC-T cell coculture. Day 9 CD25 (B). CD69 (C). and IFNAR1 (D). expression on CD4+ T cells in moDC-T cell coculture. €. Day 9 HLA-DR expression on CD4+ T cells in moDC-T cell coculture. (F). HLA-DR expression on CD4+ T cells in monocyte-T cell coculture after 72 hours. MFI represents mean fluorescence intensity. Data represent mean ± SEM from 10 or six donors. Wilcoxon tests were performed for all column pairs and corrected for multiple comparisons.

# Supplementary Figure 6 Anifrolumab pre-treatment of monocytes inhibits IFNα-mediated upregulation of activation markers HLA-ABC and HLA-DR


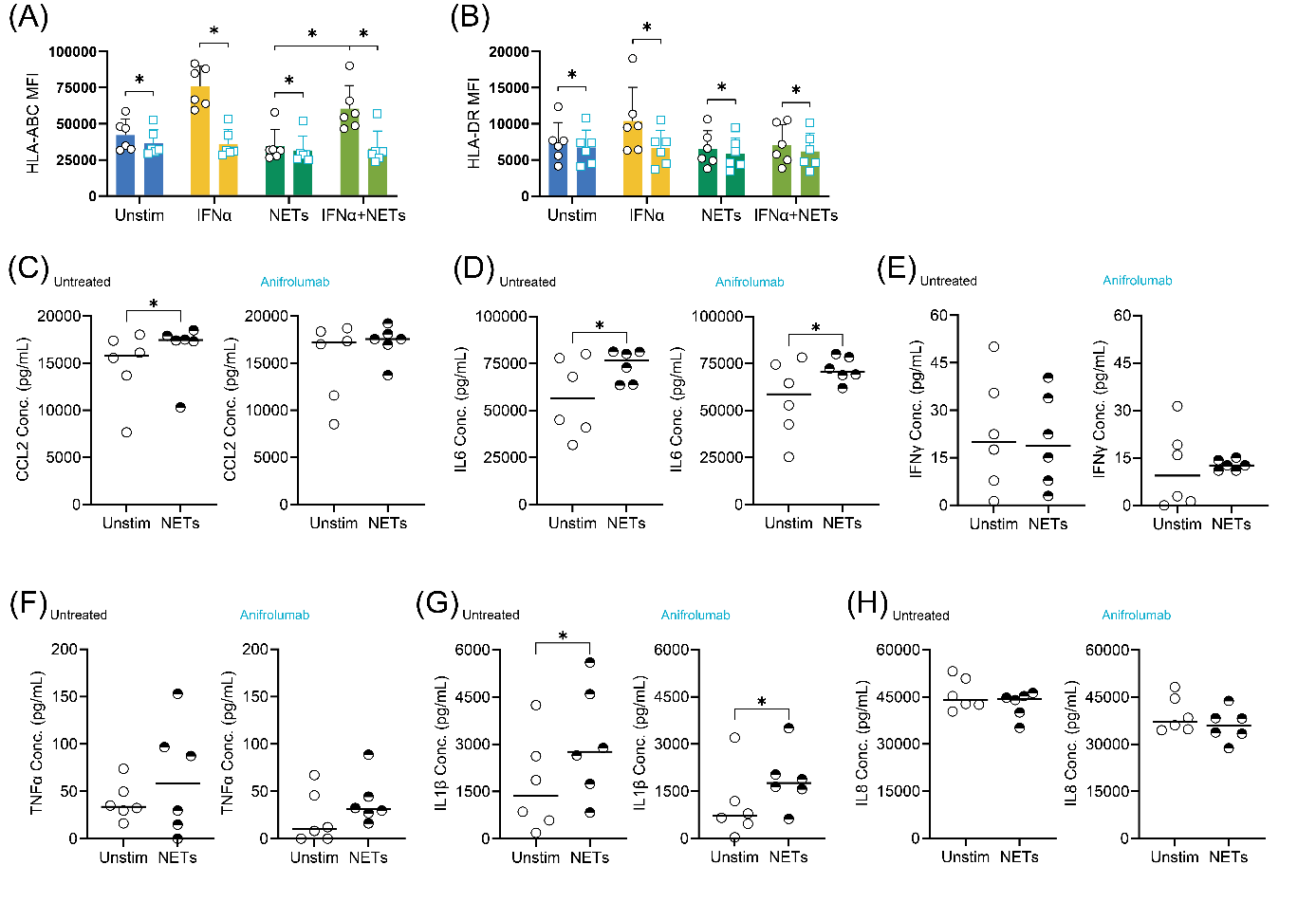


(A). HLA-ABC expression on monocytes (MFI). (B). HLA-DR surface expression on monocytes. MFI represents mean fluorescence intensity. Data represent mean ± SEM from six donors. Wilcoxon tests were performed for all column pairs and corrected for multiple comparisons for A and B. Cytokine secretion by NETs-stimulated monocytes. Monocytes were stimulated with NETs in the absence (left bars, untreated) or presence (right bars, anifrolumab treated). Panels show concentrations of (C) CCL2, (D) IL-6, (E) IFN-γ, (F) TNF-α, (G) IL-1β, and (H) IL-8 measured in culture supernatants. Data represent mean ± SEM from six donors. Wilcoxon matched-Pairs tests were used for C-H.

# Supplemental tables

**Supplemental table 1: INTERFERON cohort- seven SLE patients in remission and age- and gender-matched healthy donors.**

| HD (MDD) | Gender | Age | SLE | Gender | Age | anti-dsDNA EliA | APS | SLEDAI | prednison dose (mg) |
| --- | --- | --- | --- | --- | --- | --- | --- | --- | --- |
| 2022909 | Female | 54 | IFN254 | Female | 54 | 57.0 | Yes | 0 | 7.5 |
| 201757 | Female | 49 | IFN255 | Female | 47 | 0.8 | Yes | 0 | 0 |
| 2017374 | Female | 42 | IFN256 | Female | 42 | 47.0 | No | 0 | 0 |
| 2017220 | Female | 34 | IFN257 | Female | 31 | 30.0 | No | 2 | 7.5 |
| 2022909 | Female | 53 | IFN258 | Female | 50 | 1.5 | Yes | 0 | 10 |
| 2017459 | Female | 62 | IFN260 | Female | 61 | 0.6 | No | 0 | *0* |
| 2017524 | Male | 61 | IFN261 | Male | 58 | 13.0 | Yes | 0 | 0 |

**Supplemental table 2: PROFILE cohort- nine patients with active SLE who experienced severe flares during the two-year study.**

| Patient number | Gender | Age | Lupus nephritis in past | Medication at time of flare | Type of flare (according to definition of severe flare) |
| --- | --- | --- | --- | --- | --- |
| Pt. 01 | Female | 40 | No | Start study: Hydroxychloroquine (HCQ), azathioprine, prednisolone 5mg/day; at 9 mo stop every med except HCQ; at 12 mo start anifrolumab | New CNS involvement and new immunosuppressive therapy and  increase SLEDAI by >12 points |
| Pt. 02 | Female | 41 | No | Start study: HCQ; at 6 mo start prednisolone; at 15 mo start methotrexate; at 18 mo start tacrolimus; after 24 mo start anifrolumab. | Requiring doubling of prednisone and new immunosuppressive therapy |
| Pt. 03 | Female | 62 | Yes | Stable since start study: HCQ + azathioprine 100mg/day + prednisolone 5mg/day. | New vasculitis |
| Pt. 04 | Female | 51 | No | Start study: none. At time of flare start prednisolone + HCQ + azathioprine. At 12 mo stop prednisolone; at18 mo stop azathioprine. | New immunosuppressive therapy |
| Pt. 05 | Female | 58 | No | Start study: HCQ, ciclosporin, belimumab, prednisolone; at12 mo switch ciclosporin to methotrexate. | Increase in PGA to >2.5 |
| Pt. 06 | Female | 32 | Yes | Start study: HCQ; at 9 mo stopped HCQ; at18 mo started HCQ again. | Need for hospitalization due to SLE |
| Pt. 07 | Female | 54 | Yes | Start study: HCQ, prednisolone 5mg, azahtioprine; at 6 mo stop azathioprine, start MTX. Between 9 mo and 12 moincrease prednisolone dosage and start belimumab. At 15 mo stop MTX, decrease prednisolone dosage. | Requiring doubling of prednisone and new immunosuppressive therapy |
| Pt. 08 | Female | 62 | No | Start study: belimumab  and prednisolone 2.5mg/day. At 12 mo stopprednisolone. | Need for hospitalization due to SLE |
| Pt. 09 | Female | 52 | Yes | Start study: azathioprine,  belimumab, prednisolone 10mg/day. At 6 mo: stop aza, start MTX. At 9 mo: prednisolone dosage increased to 15mg/day. At flare between 9 mo and 12 mo stop MTX, start rituximab. At 15 mo decrease dosage prednisolone to 7.5mg/day | Need for hospitalization due to SLE and new immunosuppressive therapy |

**Supplemental table 3: Monocyte antibody panel for flow cytometry.**

| Target antigen | Fluorochrome | Cat no | Supplier |
| --- | --- | --- | --- |
| CCR5 | BB515 | 564512 | BD |
| CCL5 | perCP-cy5.5 | 515508 | Biolegend |
| viability | eFluo780 | 65-0865-18 | eBioscience |
| HLA-A.B.C | PE-Cy5 | 555554 | BD |
| CD80 | Pacific Blue | 560442 | BD |
| CD14 | V500 | 561391 | BD |
| CD86 | BV605 | 305430 | Biolegend |
| HLA-DR | BV711 | 563696 | BD |
| CD16 | BV785 | 302046 | Biolegend |

**Supplemental table 4: T cell antibody panel for flow cytometry.**

| Target antigen | Fluorochrome | Cat no | Supplier |
| --- | --- | --- | --- |
| Viability | eFluor 780 | 65-0865-18 | eBioscience |
| IFNAR1 | PE | MA5-23630 | Invitrogen |
| HLA-A.B.C* | PE-Cy5 | 555554 | BD |
| CD69 | PECy7 | 557745 | BD Pharm |
| CTV | CellTrace Violet | 10220455 | Fisher Scientific |
| CD8 | V500 | 561617 | BD Horizon |
| HLA-DR | BV605 | 562845 | BD Horizon |
| CD25 | BV711 | 563159 | BD Horizon |
| CD4 | BV785 | 300554 | Biolegend |

* HLA-A.B.C was only included for measuring T cells in moDC-T cell coculture.

**Supplemental table 5: Q-PCR primers used in this study. Forward and reverse primer sequences for target genes.**

| Target | Primer oligo | Sequence (5' to 3') |
| --- | --- | --- |
| hCCL5 | Forward | CAGTCGTCTTTGTCACCCGA |
|  | Reverse | CGGGTGGGGTAGGATAGTGA |
| hCCR5 | Forward | TTCTGGGCTCCCTACAACATT |
|  | Reverse | TTGGTCCAACCTGTTAGAGCTA |
| hCD80 | Forward | GGGAAATGTCGCCTCTCTGAA |
|  | Reverse | TCCTGGGTCTCCAAAGGTTG |
| hCD86 | Forward | TAGGTCACAGCAGAAGCAGC |
|  | Reverse | GGCAGGTCTGCAGTCTCATT |
| hRPL13A | Forward | CCTGGAGGAGAAGAGGAAAGAGA |
|  | Reverse | TTGAGGACCTCTGTGTATTTGTCAA |
