# Supplementary material for: Systemic lupus erythematosus extends beyond a type I interferonopathy, as demonstrated by NET‐activated monocytes
Source: Clin Transl Med. 2026 Feb 8;16(2):e70599. doi: 10.1002/ctm2.70599 (PMC12883034; doi:10.1002/ctm2.70599)
Supplement: Supplementary file 3 [file CTM2-16-e70599-s002.odt]

# Supplemental tables 

Supplemental table 1: INTERFERON cohort- seven SLE patients in remission and age- and gender-matched healthy donors.

| HD (MDD) | Gender | Age | SLE | Gender | Age | anti-dsDNA EliA | APS | SLEDAI | prednison dose (mg) |
| --- | --- | --- | --- | --- | --- | --- | --- | --- | --- |
| 2022909 | Female | 54 | IFN254 | Female | 54 | 57.0 | Yes | 0 | 7.5 |
| 201757 | Female | 49 | IFN255 | Female | 47 | 0.8 | Yes | 0 | 0 |
| 2017374 | Female | 42 | IFN256 | Female | 42 | 47.0 | No | 0 | 0 |
| 2017220 | Female | 34 | IFN257 | Female | 31 | 30.0 | No | 2 | 7.5 |
| 2022909 | Female | 53 | IFN258 | Female | 50 | 1.5 | Yes | 0 | 10 |
| 2017459 | Female | 62 | IFN260 | Female | 61 | 0.6 | No | 0 | 0 |
| 2017524 | Male | 61 | IFN261 | Male | 58 | 13.0 | Yes | 0 | 0 |

Supplemental table 2: PROFILE cohort- nine patients with active SLE who experienced severe flares during the two-year study.

| Patient number | Gender | Age | Lupus nephritis in past | Medication at time of flare | Type of flare (according to definition of severe flare) |
| --- | --- | --- | --- | --- | --- |
| Pt. 01 | Female | 40 | No | Start study: Hydroxychloroquine (HCQ), azathioprine, prednisolone 5mg/day; at 9 mo stop every med except HCQ; at 12 mo start anifrolumab | New CNS involvement and new immunosuppressive therapy and  increase SLEDAI by >12 points |
| Pt. 02 | Female | 41 | No | Start study: HCQ; at 6 mo start prednisolone; at 15 mo start methotrexate; at 18 mo start tacrolimus; after 24 mo start anifrolumab. | Requiring doubling of prednisone and new immunosuppressive therapy |
| Pt. 03 | Female | 62 | Yes | Stable since start study: HCQ + azathioprine 100mg/day + prednisolone 5mg/day. | New vasculitis |
| Pt. 04 | Female | 51 | No | Start study: none. At time of flare start prednisolone + HCQ + azathioprine. At 12 mo stop prednisolone; at18 mo stop azathioprine. | New immunosuppressive therapy |
| Pt. 05 | Female | 58 | No | Start study: HCQ, ciclosporin, belimumab, prednisolone; at12 mo switch ciclosporin to methotrexate. | Increase in PGA to >2.5 |
| Pt. 06 | Female | 32 | Yes | Start study: HCQ; at 9 mo stopped HCQ; at18 mo started HCQ again. | Need for hospitalization due to SLE |
| Pt. 07 | Female | 54 | Yes | Start study: HCQ, prednisolone 5mg, azahtioprine; at 6 mo stop azathioprine, start MTX. Between 9 mo and 12 moincrease prednisolone dosage and start belimumab. At 15 mo stop MTX, decrease prednisolone dosage. | Requiring doubling of prednisone and new immunosuppressive therapy |
| Pt. 08 | Female | 62 | No | Start study: belimumab  and prednisolone 2.5mg/day. At 12 mo stopprednisolone. | Need for hospitalization due to SLE |
| Pt. 09 | Female | 52 | Yes | Start study: azathioprine,  belimumab, prednisolone 10mg/day. At 6 mo: stop aza, start MTX. At 9 mo: prednisolone dosage increased to 15mg/day. At flare between 9 mo and 12 mo stop MTX, start rituximab. At 15 mo decrease dosage prednisolone to 7.5mg/day | Need for hospitalization due to SLE and new immunosuppressive therapy |

Supplemental table 3: Monocyte antibody panel for flow cytometry.

| Target antigen | Fluorochrome | Cat no | Supplier |
| --- | --- | --- | --- |
| CCR5 | BB515 | 564512 | BD |
| CCL5 | perCP-cy5.5 | 515508 | Biolegend |
| viability | eFluo780 | 65-0865-18 | eBioscience |
| HLA-A.B.C | PE-Cy5 | 555554 | BD |
| CD80 | Pacific Blue | 560442 | BD |
| CD14 | V500 | 561391 | BD |
| CD86 | BV605 | 305430 | Biolegend |
| HLA-DR | BV711 | 563696 | BD |
| CD16 | BV785 | 302046 | Biolegend |

Supplemental table 4: T cell antibody panel for flow cytometry.

| Target antigen | Fluorochrome | Cat no | Supplier |
| --- | --- | --- | --- |
| Viability | eFluor 780 | 65-0865-18 | eBioscience |
| IFNAR1 | PE | MA5-23630 | Invitrogen |
| HLA-A.B.C* | PE-Cy5 | 555554 | BD |
| CD69 | PECy7 | 557745 | BD Pharm |
| CTV | CellTrace Violet | 10220455 | Fisher Scientific |
| CD8 | V500 | 561617 | BD Horizon |
| HLA-DR | BV605 | 562845 | BD Horizon |
| CD25 | BV711 | 563159 | BD Horizon |
| CD4 | BV785 | 300554 | Biolegend |

* HLA-A.B.C was only included for measuring T cells in moDC-T cell coculture.

Supplemental table 5: Q-PCR primers used in this study. Forward and reverse primer sequences for target genes.

| Target | Primer oligo | Sequence (5' to 3') |
| --- | --- | --- |
| hCCL5 | Forward | CAGTCGTCTTTGTCACCCGA |
| Reverse | CGGGTGGGGTAGGATAGTGA |
| hCCR5 | Forward | TTCTGGGCTCCCTACAACATT |
| Reverse | TTGGTCCAACCTGTTAGAGCTA |
| hCD80 | Forward | GGGAAATGTCGCCTCTCTGAA |
| Reverse | TCCTGGGTCTCCAAAGGTTG |
| hCD86 | Forward | TAGGTCACAGCAGAAGCAGC |
| Reverse | GGCAGGTCTGCAGTCTCATT |
| hRPL13A | Forward | CCTGGAGGAGAAGAGGAAAGAGA |
| Reverse | TTGAGGACCTCTGTGTATTTGTCAA |
